# Psychosocial beliefs related to intention to use HIV testing and counselling services among suspected tuberculosis patients in Kassala state, Sudan

**DOI:** 10.1186/s12889-020-10077-w

**Published:** 2021-01-07

**Authors:** Almutaz M. Idris, Rik Crutzen, H. W. Van den Borne

**Affiliations:** 1 Department of Health Promotion, Maastricht University/CAPHRI, Maastricht, the Netherlands; 2 College of Applied Medical Science, Buraydah Colleges, Buraydah, Saudi Arabia

**Keywords:** Determinants, Beliefs, Intention, HIV testing, Suspected TB patients, Confidence interval-based estimation of relevance (CIBER)

## Abstract

**Background:**

There is limited information about the psychosocial sub-determinants regarding the use of HIV Testing and Counselling (HTC) services among suspected Tuberculosis (TB) patients in Sudan. This study aimed to assess the association between psychosocial beliefs and the intention to use HTC services and to establish the relevance of these beliefs for developing behaviour change interventions among suspected TB patients.

**Methods:**

Suspected TB patients (*N* = 383) from four separate TB facilities completed a cross-sectional questionnaire which was based on the Reasoned Action Approach theory. Eligibility criteria included attending Tuberculosis Management Units in Kassala State as suspected TB patients and aged 18–64 years. A Confidence Interval Based Estimation of Relevance (CIBER) analysis approach was employed to investigate the association of the beliefs with the intention to use HTC services and to establish their relevance to be targeted in behaviour change interventions.

**Results:**

The CIBER results showed the beliefs included in the study accounted for 59 to 70% of the variance in intention to use HTC services. The belief “My friends think I have to use HTC services” was positively associated with the intent to use HTC, and it is highly relevant for intervention development. The belief “I would fear to be stigmatized if I get a HIV positive result” was negatively related to the intention to use HTC services and was considered a highly relevant belief. The belief “If I use HTC services, health care providers will keep my HIV test result confidential” was strongly associated with the intention to use HTC services. However, the relevance of this belief as a target for future interventions development was relatively low. Past experience with HTC services was weakly associated with the intention to use HTC services.

**Conclusion:**

The intention to use HTC was a function of psychosocial beliefs. The beliefs investigated varied in their relevance for interventions designed to encourage the use of HTC services. Interventions to promote intention to use HIV testing and counselling services should address the most relevant beliefs (sub-determinants). Further study is needed to establish the relevance of sub-determinants of the intention to use HTC services for interventions development.

## Background

The Human Immunodeficiency Virus (HIV) is considered a common cause of morbidity and mortality among individuals infected with tuberculosis (TB) [1, 2]. Globally in 2018, an estimated 81 million TB cases were identified attributable to HIV infection, which accounted for 251 thousand TB deaths [3]. In Sudan, TB infection is an endemic disease, and a national TB survey in 2014, estimated the TB prevalence at 59/100,000 in the population. The incidence rate of TB patients with HIV was 2.3 per 100,000 people in 2019 [3]. The death rate of TB co-infected patients increased by 29% between 2018 and 2019 [3, 4]. Unless co-infected TB patients are diagnosed and treated early, death among them remains high.

To facilitate early detection of HIV infection among TB patients, the World Health Organization (WHO) recommends Provider-Initiated HIV Testing and Counselling (PITC) for diagnosed and suspected TB patients [5]. The PITC is a practice in which health care providers offer HIV testing and counselling routinely to all patients presenting at the health facility; patients have the right to refuse if they do not want to be tested [6]. The suspected TB patients are those patients who are presenting with symptoms and signs of TB disease (i.e., productive coughing for 2 weeks or more, fever) and for optimal patient management, accurate diagnosis is required [7]. However, in Sudan, HIV testing and counselling is offered routinely to TB patients but not to suspected TB patients. The national policy dictates that HIV testing should be provided to TB patients; a policy evident in many countries with low HIV burden due to resources implications [8].

In Sudan, the diagnosis and management of TB are provided in Tuberculosis Management Units (TBMUs) which are distributed across the country. In most TBMUs, the PITC has been introduced since 2009 [9], and co-infected patients are referred for HIV related treatment and care. TB Program data showed that annually the number of suspected TB patients attending all TBMUs in Sudan exceeds thirty thousand. Evidence from low HIV prevalence countries, such as Sudan has indicated that the prevalence of HIV infection in suspected TB patients is high [10, 11]. The same is observed in African countries with a high reported HIV burden [12, 13].

Routine HIV testing of suspected TB patients offers an opportunity for early detection of HIV and treatment, and this is associated with decreased morbidity and mortality [14]. However, there are no data on the HIV testing rates of Sudanese suspected TB patients.

Different factors can influence the suspected TB patients’ decision to test or not test for HIV at their initial encounter with TBMUs, including lack of knowledge about HIV testing, no previous experience for HIV testing [15] confidentiality of HIV testing [16], training of health care provider and availability of HIV testing guidelines [13].

Also, growing evidence suggests that HIV testing and counselling behaviour is predicted by social-cognitive factors such as subjective norms [17–19], attitude [20, 21], perceived behavioural control and intention [21, 22] regarding HIV testing, also risk perception [23–26] and perceived susceptibility of HIV infection [19].

Social cognitive theories are useful in understanding the (sub-) determinants of HIV testing behaviour [21, 22] and for developing an intervention to change this behaviour [27, 28]. Applying these theories to behaviour change programs may increase their potential to change HIV testing behaviour.

The Reasoned Action Approach (RAA) [29] is one of these theories and proposes that intention is the most proximate determinant of performing a particular behaviour. The intention is predicted by three sub-determinants, including attitude, which is about evaluating the benefits of performing the behaviour [29], subjective norms, or the social pressure on the person to do or not to do the behaviour, and perception of control over doing the behaviour [30, 31]. The attitude, subjective norms and perceived behavioural control are a function of behavioural beliefs, normative beliefs, and control beliefs, respectively [30, 32–34]. Also, the RAA can be extended to include other factors which may influence engaging in HIV testing behaviour such as HIV risk perception and past experiences [35].

There is a lack of information on psychosocial sub-determinants regarding HIV testing behaviour among suspected TB patients in Sudan. This study employed the RAA to assess the psychosocial beliefs that are associated with the suspected TB patients’ intention to use HTC services and to assess the relevance of these beliefs for selection for interventions to enhance that intention among study group in Kassala State, Sudan.

## Methods

The study follows the STROBE Statement for reporting observational studies [36] guidelines.

### Study design and settings

A cross-sectional study design was applied, and data were collected from July 2017 to February 2018 among suspected TB patients in Kassala State. The Kassala State is one of the eighteen states of Sudan, situated in the eastern region of the country. The State is divided into eleven localities and is covering an area of 42,282 km^2^. In 2018, the population of the State was estimated at 2.5 million based on the 2008 population census. According to the State TB program, the prevalence of TB was about 71 per 100,000 population in 2018. The TB/HIV burden among TB patients appears to be high, with an estimated 18.6% of the TB patients being infected with HIV [9]. At the time of the study, there were twenty-two TBMUs providing diagnostic services and treatment for suspected TB patients. All TBMUs offered HTC services.

### Study population and recruitment

A three-sampling stage was performed. The first stage was simple random sampling to select four localities from the sampling frame of eleven localities in Kassala State. The second stage was a random selection of one TBMU from each selected locality. A total of four TBMUs were selected. The third was on-site systematic sampling for selection of participants from each TBMU where the first participant was randomly selected from the clinic registers, and then every third was interviewed using a constant numeric interval. The number of selected participants for sampling from each TBMU was proportional to the number of suspected TB patients attending at that TBMU. Any participant who refused to participate for any reason was replaced by the immediate next one until we reached the required sample size. All the suspected TB patients who were attending TBMUs for diagnosis purposes during the study period were eligible for this study. We included patients suspected of TB who aged 18–64 years in the selected TBMUs. A sample size of 383 participants was computed by sample estimation for correlation with pre-specified 95% confidence interval [37]. This sample size allows estimation of a correlation of .05 with a width of.10.

### Study variables

The independent variable of this study was the intention to use HTC services in TBMUs in Kassala State in the next 3 months. The explanatory variables were behavioural beliefs, normative beliefs, and control beliefs regarding the use of HTC services in the TBMUs in the next 3 months, risk perception of HIV infection, and exposure to HTC services during the last year. Information on demographic and socioeconomic variables, including age, gender, residence, marital status, education, working, HIV and HTC related-knowledge, were also collected.

### Data collection and measurements

Trained data collectors used a structured questionnaire to collect data from the participants. All respondents provided consent to participate before included in the study. Uneducated participants were interviewed, and educated participants fill out the questionnaire by themselves.

The questionnaire was based on the RAA [29]. The statements used to measure the beliefs in this study were developed from a literature review [38–42] and elicitation study conducted in the study population. The elicitation study is essential to identify beliefs [43] that associated with the intention to use HTC services.

The intention to use HTC services was measured by three items which were: (1) I intent, to use HTC services in the next 3 months, (2) I expected to use HTC services in the next 3 months and (3) I want to use HTC services in the next 3 months, and were each measured on a 7–point Likert scale. The higher score indicated a more positive intention.

The behavioural beliefs were measured by asking the participants to rate five statements regarding using HTC services in the next 3 months. The statements were: (1) “If I use the HTC services I will know my HIV status”, (2) “My using HTC services facilitates my treatment if I have a HIV positive test result”, (3) “If I use the HTC services I could prevent infecting my family from HIV infection”, (4) “If I use the HTC services I would feel worries about my HIV test result”, and (5) “If I use the HTC services I would have information about HIV infection”. The participants replied on a seven-point Likert scale ranging from 1(unlikely) to 7 (likely).

The normative beliefs were evaluated by using four items which were answered on a seven-point Likert scale (with disagree (1) and agree (7) as anchors). The four items were: (1) “My doctor thinks I should use the HTC services in the next three months”, (2) “My friends think I have to use the HTC services in the next three months”, (3) “My partner thinks I should use HTC services in the next three months.”, and (4) “My parent thinks I should use HTC services in the next three months.”

Five items assessed the control beliefs: (1) “Health care providers keep my HIV test result confidential”, (2) “I would fear to be stigmatized if I get a HIV positive test result”, (3) “I would fear losing my partner if I have HIV positive test result”, (4) “I would find it difficult to disclose my HIV positive test result to others”, and (5) “I could have the cost to reach HTC services”. Responses were on a seven-point unipolar (unlikely (1) -likely (7)) scale.

Two items assessed the perceived HIV risk: “I think I may be infected with the HIV infection”, and “I think my partner may be infected with the HIV infection”, scored on a seven-point (disagree (1)-agree (7)) scale.

Previous exposure to HTC services was measured by one item:“ In the last year I had an experience with HTC services”. Responses ranged from disagree (1) to agree (7).

HIV and HTC related knowledge was assessed by five questions adapted from previous studies [44–46]. The question were**:** (1) healthy-looking person can be infected with HIV, (2) HIV infection transmitted through sexual intercourse, (3) HIV infection can be prevented by using a condom, (4) HTC is provided at TB facilities, and (5) HTC is important for TB and suspect TB patients. Questions were answered using ‘Yes’, ‘No’ and ‘Do not know’. Yes, answer coded 1 and No or Do not know code as 0.

### Statistical analyses

Data analyses were conducted by R version 3.6.1 [47] and Statistical Package for Social Sciences (SPSS) version 21. Descriptive statistics of the participants’ demographic and socioeconomic characteristics were presented as frequencies and proportions. Averages were calculated for age and knowledge about HIV and HTC services. The relevance of all studied beliefs of the intention to use HTC was assessed by performing Confidence Interval Based Estimation of Relevance (CIBER) analysis [48].

The CIBER is a data visualization method which presents different information on a diamond plot to facilitate selection of the sub-determinants for intervention development. The diamond plot is divided into a left-hand panel and right-hand panels with diamonds. One diamond shape in the left-hand panel represents both the means of the sub-determinants (in this study, the beliefs regarding the use of the HTC services) and its 99.99% confidence interval. While each diamond in the right-hand panel presents the associations (e.g., correlation) between the beliefs and the outcome variable (In this study the intention to use HTC services) with a 95% confidence interval. The dots around the left-hand panel diamonds are all the participants’ item scores. The question used to assess each belief with its anchors is shown on the side of the left-hand panel. At the top of the plot, the confidence interval of the explained variance (R^2^) in the intention to use HTC services based on beliefs that are included in the analysis is provided.

## Results

Three hundred and eighty-three suspected TB patients are included in the study. The descriptive statistics of the study respondents are presented in Table 1. The mean age of the participants was 41.2 (± 11.6) years, and 221 (58%) were male. Among the study participants, 16.4% were in the age group 45 to 49 years, and 44.1% were married. Of study participants, 29.5% were from Kassala Locality, 27.4% from New Halfa Locality, 24.5% from Khashm Elgriba locality, and 18.5% from Wad Elheliw Locality. One hundred and forty-three (37.3%) of the participants were illiterate, 151 (39.4%) reported having received primary education, and 89 (23.2%) said they had secondary or higher education. The majority of the participants were unemployed, 17% were employed, and 29% were self-employed. The mean score of knowledge about HIV infection and HTC services of the participants was 3.2 (± 1.2).

**Table 1:** Demographic and Socioeconomic characteristics of the study participants (*N* = 383)

Variables	Number (%)
**Gender**	
Male	221(58%)
Female	162 (42.3%)
**Age Group**	Mean 41.2 (± 11.6)
< 25 years	36 (9.4%)
25–29 years	42 (10.9%)
30–34 years	39 (10.3%)
35–39 years	49 (12.8%)
40–44 years	59 (15.4%)
45–49 years	63 (16.4%)
50–54 years	64 (16.7%)
≥ 55 years	31(8.1%)
**Marital status**	
Married	169 (44.1%)
Divorce	63 (16.4%)
Widow	47 (12.3%)
Single	104 (27.2%)
**Education level**	
Illiterate	143 (37.3%)
Primary	151(39.4%)
Secondary or higher	89 (23.2%)
**Occupation Status**	
Unemployed	204 (53.3%)
Employed workers	68 (17.7%)
Self-employed workers	111 (28.9%)
**Residence per localities**	
Kassala Locality	113 (29.5%)
New Halfa Locality	105 (27.4%)
WadElheliw Locality	71(18.5%)
Khashm Elgriba Locality	94(24.5%)
Knowledge about HIV infection and HTC services	Mean 3.2 (± 1.2)

**Fig. 1:**
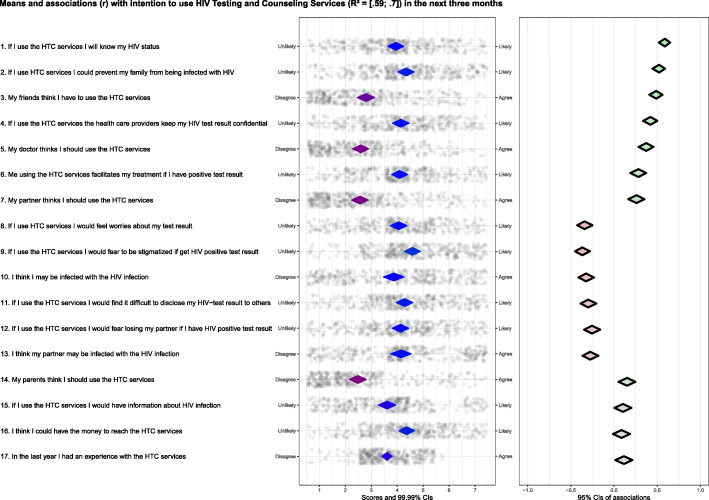
CIBER plot of psychosocial beliefs of the intention to use HTC services and past HTC experience of TB suspect patients in Kassala State (*N* = 383)

Figure 1 presents the results of the CIBER analysis. All beliefs included in the study explained 59 to 70% of the variance in the intention to use HTC services among suspected TB patients. The results show that participants’ beliefs regarding knowing their HIV status if they use HTC services scored in the middle of the scale, and it had a strong positive relationship with the intention to use HTC services. The mean of the participants who think that “health care providers keep their HIV test results confidential” was in the mid of the scale, and this belief had a positive and strong association with the intention to use HTC services. Also, the mean of the participants’ belief that “Using HTC services could prevent infecting their family from HIV infection” was roughly in the middle of the panel and had a strong positive association with the intention to use HTC services. The item scores of the belief “My friends think I have to use the HTC services” was relatively low; however, this belief was strongly associated with the intention. The belief “My doctor thinks I should use the HTC services” was positively associated with the intention to use HTC services and had a low mean score. The mean score of the belief “My using HTC services facilitates my treatment if I have HIV positive test result” was relatively on the middle of the range, and this belief was strongly and positively associated with the intention to use HTC services.

On average, participants believed that if they use the HTC services, they would feel worried about their HIV test results. However, this belief was negatively associated with the intention to use HTC services. The participants’ belief regarding the “fear to be stigmatized if they get a HIV positive test result”, scored in the middle of the scale and was negatively associated with the intention to use HTC services. The item scores of the beliefs “I think my partner or I may be infected with HIV infection” were negatively associated with intention to use HTC services and their means were in the middle of the scale. All participants who believed to have “difficulties in disclosing their HIV positive test result” scored in the middle of the scale and this belief was negatively associated with the intention to use HTC services.

As Fig. 1 shows, the mean score of the belief “If I use HTC services I would fear losing my partner if I have HIV positive test result” was in the middle of the scale and was negatively associated with the intention to use HTC services.

Also, our results show that the mean score of the belief “My parent thinks I should use HTC services” among the participants was relatively low and it was weakly associated with the intention to use HTC services. The item “In the previous year, I had an experience with HTC services” had a weak association with intention to use HTC services and its mean score was in the middle of the scale .

The participants believe that by using HTC services, they would “have information about HIV infection” scored in the centre of the scale and was not associated with the intention. The participants had a low average score regarding the costs to reach the HTC services, and the belief was not associated with the intention to use HTC services.

## Discussion

Previous studies on suspected TB patients have focused on assessing the prevalence of HIV infection [12, 13] and the feasibility [11] of HIV testing rather than investigating the psychosocial (sub-) determinants influencing the use of HTC services. The purpose of the study was to assess the association between psychosocial beliefs and the intention to use HTC services among suspected TB patients in Kassala State, Sudan. In addition, we assessed the relevance of psychosocial beliefs in developing interventions to enhance HTC service-related intention among this group.

The findings of our study suggest that several psychosocial beliefs seem to influence the intention of the suspected TB patients to use HTC services and they vary in terms of their relevance to possible interventions promoting this intention among this group. In general, the variance in the intention to use HTC services explained by all beliefs included in our study ranged from 59 to 70%. This explained variance is higher in comparison to previous studies [39, 49].

Our analysis (Fig. 1) suggests a significant positive association between the intention to use HTC and the participants’ belief “If they use the HTC services, I will know my HIV status”. This means that Sudanese suspected TB patients who hold this belief are more likely to intend to use HTC services. The mid-scale scores for this belief indicate that almost half of the participants are already convinced that use of HTC services results in knowing their HIV status, and this may be due to influence of previous exposure to HIV related interventions. The combination of the mid-scale belief scores and its strong positive association with the intention to use HTC services makes it a relatively low relevant belief for intervention. A previous study [50] from a different setting suggests that learning one’s HIV status is a reason for accepting HIV testing.

This study also, demonstrates a strong positive relationship between the belief “My friends think I have to use HTC services” and the intention to use HTC services. This association suggests that the perception of suspected TB patients of what their friends think may have a great influence on their intention to utilize HIV counselling and testing services. The finding may be compared with the result of a previous study in which the effect of peer pressure on HIV testing was reported among adolescent [17]. The observed low mean of the belief’s scores is another significant finding as it indicates that a large proportion of the study participants did not believe that their friends think they should do so. These findings make the belief a high relevant belief for intervention and suggest that the intention to use HTC services may be improved by behaviour change interventions that target the interpersonal environment of suspected TB patients.

The belief “My using the HTC services facilitates my treatment if I have a positive test result” was identified to be positively associated with the intention to use HTC services. Previous research [51] found that the availability of HIV treatment may influence the willingness of the individuals to test for HIV. In term of intervention development, this belief is not a highly relevant belief due to the fact that the mean score in the middle of the scale. The mid-scale mean indicates that respondents are already convinced that using HTC services would help them to receive treatment if they were infected with HIV. This implies that this belief needs to be sustained or tailored to an intervention message to target those who are not convinced that HTC services assist them to have treatment if they have a HIV infection.

The belief “If I use the HTC services, I could prevent infecting my family from HIV infection” was significantly associated with the intention to use HTC services. However, the relevance is low because the scores are in the middle of the scale, which indicates that most of the participants are convinced that by using HTC services they could prevent their families from HIV infection. Concern for protecting the family against HIV as an important motiving factor in the use of HTC services was documented in a study among married individuals in Tanzania [21].

Our study finds a strong association between the belief “If I use the HTC services health care providers keep my HIV test result confidential” and the intention to use HTC services. The combination of strong association and response scores distributed around the middle of the scale means this belief is of relatively low relevance. Interventions that increase or reinforce the belief in the confidentiality of the HIV test and test result are likely to improve the intention to utilize HTC services [52–54]. The confidentiality is influenced by the characteristics of the HIV testing service providers [55]. Lack of training of HTC services providers and HIV testing guidelines may result in compromising the confidentiality of HIV test and test result [55]. In another study [13] training of health providers and the availability of testing and counselling guidelines were suggested to mitigate the fear related to breach of the confidentiality of test and testing result.

According to our study findings, the belief “If I use the HTC services I would fear to be stigmatized if I get a HIV positive test result” was found to be strongly and negatively associated with the intention to use HTC services. Response scores are in the upper part of the scale, which indicates that most of the participants believe that HIV infection is linked to stigma. Among Sudanese communities, HIV infection is commonly associated with stigma and discrimination [56]. These findings suggest that this belief is highly relevant and imply a need for change. Other studies describing stigma as a barrier for HIV testing [20, 57, 58] have been carried out in different settings.

Our study shows that the belief “I think I may be infected with HIV infection” was significantly and negatively associated with the intention to use HTC services. Participants’ scores are in the middle of the panel suggests that about half of the participants convinced that they might not be infected with HIV. Most individuals in Sudan perceive themselves as not at risk of HIV infection [56], and this could be attributed to the low prevalence of HIV infection among the general population in the country [59]. However, the combination of the negative association and the middle scale scores make this belief a low relevant intervention target. A study among pregnant women reported that perception of susceptibility to HIV infection might be a reason to test for HIV [60].

The study demonstrates a weak correlation between the participants’ previous experience concerning HTC, and the participants’ scores are in the middle of the panel, indicating that quite a number of them had no prior experience. The past experience represents particular knowledge that individuals hold about the behaviour, and it was found to be a predictor of behavioural intentions [61]. A previous study among suspected TB patients suggested that past HIV testing and counseling experience may influence the uptake of HIV testing services [15]. However, the combination of the weak association and middle scale scores indicate a low relevance of the previous experience related beliefs for intervention development for our target group.

A number of limitations are present in our study; first, the use of a cross-sectional study design limited ability to establish causality. Future prospective research would be useful to establish causality. Secondly, the study focused on the intention to use HTC services rather than the actual use of the services. Therefore, future research on actual behaviour is needed. The third limitation was the self-reporting of all beliefs in the study and the intention in the study. However, explaining the study objectives to all participants and ensuring anonymity and confidentiality are expected to encourage them to provide honest and accurate responses.

The fourth limitation was that the participants with no education were interviewed in person so that they may have provided more socially acceptable answers. The familiarity of our data collectors with participants’ characteristics and their understanding of the study objectives was anticipated to reduce this limitation.

Despite these limitations, our study findings highlight some relevant beliefs for developing an intention enhancing intervention among suspected TB patients in Sudan and other countries with similar epidemiological and social contexts. Also, it provides a base for future researches on establishing the relevance of the sub-determinants to be targeted with interventions to promote the use of HTC services.

## Conclusion

The psychosocial beliefs are important in understanding the intention to use HTC services and in developing interventions to enhance that intention. The current study findings suggest that the intention to use HIV testing and counselling services was a function of different beliefs. The relevance of the beliefs for intervention development to enhance the intention to use HTC services differed. The beliefs “My friends think I have to use the HTC services” and “fear to be stigmatized if I get a HIV positive test result” were associated with intention and highly relevant beliefs for interventions development. However, the relevance of the beliefs of “confidentiality of test result” and “perceived personal risk for HIV infection” were relatively low. The belief “I will know my HIV status” was associated with the intention to use HTC services, but it was a low relevant belief for intervention development. Past experience of HTC services was weakly associated with intention to use HIV testing and counselling services. Interventions targeted at promoting the intention to use HIV testing and counselling services should address the most relevant beliefs (sub-determinants). Further study is needed to establish the relevance of sub-determinants of the intention to use HTC services.

## Supplementary Information

**Additional file 1.**

## Data Availability

The datasets of this study are available from the corresponding author on request.

## Abbreviations

AIDS: Acquired Immune Deficiency Syndrome
CIBER: Confidence Interval Based Estimation of Relevance
HIV: Human Immunodeficiency Virus
HTC: HIV Testing and Counselling
PITC: Provider-initiated HIV testing and counselling
RAA: Reasoned Action Approach
STROBE: STrengthening the Reporting of OBservational studies in Epidemiology
TB: Tuberculosis
TBMUs: Tuberculosis Management Units
WHO: World Health Organization
