# Supplementary material for: Psychosocial beliefs related to intention to use HIV testing and counselling services among suspected tuberculosis patients in Kassala state, Sudan
Source: BMC Public Health. 2021 Jan 7;21:75. doi: 10.1186/s12889-020-10077-w (PMC7791737; doi:10.1186/s12889-020-10077-w)
Supplement: Supplementary file 1 — Additional file 1. [file 12889_2020_10077_MOESM1_ESM.docx]

**Questionnaire of psychosocial determinants of intention to use HTC testing behavior among TB suspected patients attending Tuberculosis Management Units in Kassala State, Sudan**

***[Translated to English; original version in Arab]***

**Informed Consent Form**

My name is_______ I work for _________ and I am interviewing people here (Place name) to obtain their beliefs and views that are associated with using HIV testing and counseling (HTC) services that are offered to in Tuberculosis management Units antenatal in Kassala State.

You have been selected as a respondent. We would like to ask you about your beliefs and views regarding HIV testing and Counseling (HTC) services in TB facilities. This will help us to have better understanding about the determinants of HTC behavior among TB suspected patients like you. We would like you to know that there is no right or wrong answers for these questions you will be asked. Participation in the study is voluntary,which means that if you choose not to participate, your treatment and care will not be affected. Not participating is not associated withpenalties, also your relations with Health Facility will not be affected.

Ifyou decide to participate, you can ask any question about anything at any time ifyou feel there is something not clear to you. You can discontinue participation in the study at any time. Also, your name will not be written anywhere, and will never be used with any information you provide.

In addition, your answers will be held in strict privacy and confidentiality and will use only for improving HTC services purposes. This questionnaire will take about 30 to 40 minutes.

Would you like to participate in the study? [If not, thank the respondent for his time.]

| **personal Data** | | |
| --- | --- | --- |
| No: | Question | Answer |
| Q1 | Age? |  |
| Q2 | Sex? |  Female  Male |
| Q3 | Residence per locality ? | Write the locality name ___________________________ |
| Q4 | Education level? |  Illiterate/Khalwa  Primary school  Secondaryschoolor higher |
| Q5 | Occupation?(Write) |  |
| Q6 | Marital status? |  Married DivorceSingle  Widow |

| **Target:** Intention of suspected TB patients to use HIV testing and Counseling (HTC) services in TB facilities in the next 3 months in Kassala State |
| --- |

| **Section1**:  **Direct measures** of Intention, Attitude, Perceived Social Norms and Perceived behavior control |
| --- |

| Extremely un likely | Un likely | Slightly  Un likely | Not sure | Slightly Likely | Likely | Extremely Likely | **Intention**: please rate to what extent you think the below statements are likely or unlikely for you? | |
| --- | --- | --- | --- | --- | --- | --- | --- | --- |
| 🞎 | 🞎 | 🞎 | 🞎 | 🞎 | 🞎 | 🞎 | I intend to use HIV testing and counseling services at TB facility in Kassala State in the next 3 months? | Q7 |
| 🞎 | 🞎 | 🞎 | 🞎 | 🞎 | 🞎 | 🞎 | I expect to use HIV testing and counseling services at TB facility in Kassala State in the next 3 months? | Q8 |
| 🞎 | 🞎 | 🞎 | 🞎 | 🞎 | 🞎 | 🞎 | I want to use HIV testing and counseling services at TB facility in Kassala State in the next 3 months? | Q9 |

| Extremely unimportant | Unimportant | Slightly unimportant | Not sure | Slightly important | important | Extremely important | **Attitude1:** please rate to what extent you think the below statement are important or un important to you? | |
| --- | --- | --- | --- | --- | --- | --- | --- | --- |
| 🞎 | 🞎 | 🞎 | 🞎 | 🞎 | 🞎 | 🞎 | My using HIV Testing and Counseling services at TB facility in Kassala State in the next 3 months would be | Q10 |

| Extremely un pleasant | Un pleasant | Slightly Un pleasant | Not sure | Slightly Pleasant | Pleasant | Extremely Pleasant | **Attitude2:**please rate to what extent you think the below statement is pleasant or unpleasant for you | |
| --- | --- | --- | --- | --- | --- | --- | --- | --- |
| 🞎 | 🞎 | 🞎 | 🞎 | 🞎 | 🞎 | 🞎 | My using HIV Testing and Counseling services at TB facility in Kassala State in the next 3 months would be | Q11 |

| Extremely bad | Bad | Slightly Bad | Not sure | Slightly Good | Good | Extremely Good | **Attitude 3**: please rate to what extent you thought the below statement is good or bad for you | |
| --- | --- | --- | --- | --- | --- | --- | --- | --- |
| 🞎 | 🞎 | 🞎 | 🞎 | 🞎 | 🞎 | 🞎 | My using HIV Testing and Counseling services at TB facility in Kassala State in the next 3 months would be | Q124 |

| Extremely Disagree | Disagree | Slightly Disagree | Not sure | Slightly Agree | Agree | Extremely Agree | **Subjective Norms**: please rate to what extent you agree or disagree with below statement s (select one answer) | |
| --- | --- | --- | --- | --- | --- | --- | --- | --- |
| 🞎 | 🞎 | 🞎 | 🞎 | 🞎 | 🞎 | 🞎 | Most people who are important to me appreciate my using of HTC services in the next 3 months in TB facilities | Q13 |
| 🞎 | 🞎 | 🞎 | 🞎 | 🞎 | 🞎 | 🞎 | Most people who are important to me encourage me to use HTC services in the next 3 months in TB facilities | Q14 |
| 🞎 | 🞎 | 🞎 | 🞎 | 🞎 | 🞎 | 🞎 | Most people who are important to me use HTC services in TB facilities | Q15 |
| 🞎 | 🞎 | 🞎 | 🞎 | 🞎 | 🞎 | 🞎 | Most people like me use HTC services in TB facilities | Q16 |
| 🞎 | 🞎 | 🞎 | 🞎 | 🞎 | 🞎 | 🞎 | TB group members in Kassala State support me to use HTC service in TB facilities in Kassala State | Q17 |

| Extremely Disagree | Disagree | Slightly Disagree | Not sure | Slightly Agree | Agree | Extremely Agree | **Perceived Behavior Control**: Please rate to what extent you agree or disagree with below statements | |
| --- | --- | --- | --- | --- | --- | --- | --- | --- |
| 🞎 | 🞎 | 🞎 | 🞎 | 🞎 | 🞎 | 🞎 | I feel I would be capable to use HTC service in TB facilities during the next 3 months in Kassala State if I wanted | Q19 |
| 🞎 | 🞎 | 🞎 | 🞎 | 🞎 | 🞎 | 🞎 | I am confident that I could use HTC service in TB facilities during the next 3 months in Kassala State | Q20 |
| 🞎 | 🞎 | 🞎 | 🞎 | 🞎 | 🞎 | 🞎 | Whether I use HTC service in TB facilities in the next 3 months or not is entirely up to me | Q21 |

| **Section 2**:Indirect measures of Reasoned Action Approach constructs | | | | | | | | |
| --- | --- | --- | --- | --- | --- | --- | --- | --- |
| Extremely un likely | Un likely | Slightly Un likely | Not sure | Slightly Likely | Likely | Extremely Likely | **A.Attitudinal behavioralbelief strength:** please rate towhat extent you think the below statements are likely or unlikely if you use HTC services in TBfacilities in Kassala state in the next 3month? | |
| 🞎 | 🞎 | 🞎 | 🞎 | 🞎 | 🞎 | 🞎 | I will know my HIV sero-status | Q22 |
| 🞎 | 🞎 | 🞎 | 🞎 | 🞎 | 🞎 | 🞎 | Facilitatesmy treatment if I have positive test result | Q23 |
| 🞎 | 🞎 | 🞎 | 🞎 | 🞎 | 🞎 | 🞎 | I would feel worried about HIV test result | Q24 |
| 🞎 | 🞎 | 🞎 | 🞎 | 🞎 | 🞎 | 🞎 | I could prevent infecting my family from HIV infection | Q25 |
| 🞎 | 🞎 | 🞎 | 🞎 | 🞎 | 🞎 | 🞎 | I would have information about HIV infection | Q26 |
| 🞎 | 🞎 | 🞎 | 🞎 | 🞎 | 🞎 | 🞎 | Have health care providers whom you would like | Q27 |

| Extremely Good | Good | Slightly Good | Not sure | Slightly Bad | Bad | Extremely Bad | **B. Outcome evaluation:**please rate to what extent you think the below statements are very good or very bad if you use HTC services in TB facilities in Kassala state in the next 3 month? | |
| --- | --- | --- | --- | --- | --- | --- | --- | --- |
| 🞎 | 🞎 | 🞎 | 🞎 | 🞎 | 🞎 | 🞎 | Me know my HIV sero-status is | Q28 |
| 🞎 | 🞎 | 🞎 | 🞎 | 🞎 | 🞎 | 🞎 | Facilitating my treatment if I have HIV positive result is | Q29 |
| 🞎 | 🞎 | 🞎 | 🞎 | 🞎 | 🞎 | 🞎 | My worries waiting for HIV test result is | Q30 |
| 🞎 | 🞎 | 🞎 | 🞎 | 🞎 | 🞎 | 🞎 | Me prevent infecting my family from HIV infection is | Q31 |
| 🞎 | 🞎 | 🞎 | 🞎 | 🞎 | 🞎 | 🞎 | Having information about HIV infection is | Q32 |
| 🞎 | 🞎 | 🞎 | 🞎 | 🞎 | 🞎 | 🞎 | Me having health care providers whom I like is | Q33 |

| Extremely Disagree | | Disagree | | Slightly Disagree | | Not sure | | Slightly Agree | | Agree | | Extremely Agree | | **C. Normative beliefs Strength and Motivation to comply (MC):** please rate to what extent you agree or disagree with below statements | | | |
| --- | --- | --- | --- | --- | --- | --- | --- | --- | --- | --- | --- | --- | --- | --- | --- | --- | --- |
| 🞎 | | 🞎 | | 🞎 | | 🞎 | | 🞎 | | 🞎 | | 🞎 | | My doctor thinks I should use HTC service in TB facilities in the next 3 months in Kassala State? | | Q34 | |
| 🞎 | | 🞎 | | 🞎 | | 🞎 | | 🞎 | | 🞎 | | 🞎 | | I want to do what my Doctor thinks I should do? | | Q35 | |
| 🞎 | | 🞎 | | 🞎 | | 🞎 | | 🞎 | | 🞎 | | 🞎 | | My counselor thinks I should use HTC service in TB facilities during the next 3 months in Kassala State? | | Q36 | |
| 🞎 | | 🞎 | | 🞎 | | 🞎 | | 🞎 | | 🞎 | | 🞎 | | I want to do what my counselor thinks I should do? | | Q37 | |
| 🞎 | | 🞎 | | 🞎 | | 🞎 | | 🞎 | | 🞎 | | 🞎 | | My friends think I have to use HTC services in TB facilities during the next 3 months in Kassala State? | | Q38 | |
| 🞎 | | 🞎 | | 🞎 | | 🞎 | | 🞎 | | 🞎 | | 🞎 | | I want to do what my friends thinks I should do? | | Q39 | |
| 🞎 | | 🞎 | | 🞎 | | 🞎 | | 🞎 | | 🞎 | | 🞎 | | My partner thinks I have to use HTC services in TB facilities during the next 3 months in Kassala State? | | Q40 | |
| 🞎 | | 🞎 | | 🞎 | | 🞎 | | 🞎 | | 🞎 | |  | | I want to do what my partner thinks I should do? | | Q41 | |
| 🞎 | | 🞎 | | 🞎 | | 🞎 | | 🞎 | | 🞎 | | 🞎 | | My parents think I should use HTC service in TB facilities during the next 3 months in Kassala State? | | Q38 | |
| 🞎 | | 🞎 | | 🞎 | | 🞎 | | 🞎 | | 🞎 | | 🞎 | | I want to do what my parent thinks I should do? | | Q42 | |
| Extremely un likely | Un likely | | Slightly Un likely | | Not sure | | Slightly Likely | | Likely | | Extremely Likely | | **D. Control belief strength (CS):**please rate to what extent thebelow statements are likely or unlikely to get tested in TB facilities in Kassala state in the next 3 month? | | | |  |
| 🞎 | 🞎 | | 🞎 | | 🞎 | | 🞎 | | 🞎 | | 🞎 | | I could have the cost to reach HTC services | | Q43 | |  |
| 🞎 | 🞎 | | 🞎 | | 🞎 | | 🞎 | | 🞎 | | 🞎 | | Health care providers will keep my HIV test result confidential? | | Q44 | |  |
| 🞎 | 🞎 | | 🞎 | | 🞎 | | 🞎 | | 🞎 | | 🞎 | | I would find it difficult to disclose my HIV positive test result to others | | Q45 | |  |
| 🞎 | 🞎 | | 🞎 | | 🞎 | | 🞎 | | 🞎 | | 🞎 | | I would fear to be stigmatized if I get HIV positive result | | Q46 | |  |
| 🞎 | 🞎 | | 🞎 | | 🞎 | | 🞎 | | 🞎 | | 🞎 | | I would fear losing my partner if I infected with HIV infection | | Q47 | |  |
| 🞎 | 🞎 | | 🞎 | | 🞎 | | 🞎 | | 🞎 | | 🞎 | | You will have fear people assume you are HIV positive? | | Q48 | |  |

| Extremely Easier | Easier | Slightly Easier | Not sure | Slightly Difficult | Difficult | Extremely difficult | E. Power of Control belief (PB): please rate towhat extent the presence of below statements make your use of HTC services in TB facilities in Kassala state in the next 3 month easier of difficult? | |
| --- | --- | --- | --- | --- | --- | --- | --- | --- |
| 🞎 | 🞎 | 🞎 | 🞎 | 🞎 | 🞎 | 🞎 | Having the cost to reach HTC services | Q49 |
| 🞎 | 🞎 | 🞎 | 🞎 | 🞎 | 🞎 | 🞎 | HTC health care providers keep my HIV test result confidential? | Q50 |
| 🞎 | 🞎 | 🞎 | 🞎 | 🞎 | 🞎 | 🞎 | Having difficulty to disclose your HIV positive result to others | Q51 |
| 🞎 | 🞎 | 🞎 | 🞎 | 🞎 | 🞎 | 🞎 | Fear to be stigmatized if I get a HIV positive result | Q52 |
| 🞎 | 🞎 | 🞎 | 🞎 | 🞎 | 🞎 | 🞎 | My fear of losing my partner if I infected with HIV infection | Q53 |
| 🞎 | 🞎 | 🞎 | 🞎 | 🞎 | 🞎 | 🞎 | My having fear of people assuming I am HIV positive? | Q54 |

| Extremely Disagree | Disagree | Slightly Disagree | Not sure | Slightly Agree | Agree | Extremely Agree | **Perceived susceptibility**: Please rate to what extent you agree or disagree with below statements (select only one answer) | |
| --- | --- | --- | --- | --- | --- | --- | --- | --- |
| 🞎 | 🞎 | 🞎 | 🞎 | 🞎 | 🞎 | 🞎 | I think I myself maybe infected with HIV infection | Q55 |
| 🞎 | 🞎 | 🞎 | 🞎 | 🞎 | 🞎 | 🞎 | I think mypartner maybe infected with HIV infection | Q56 |

| Extremely Disagree | Disagree | Slightly Disagree | Not sure | Slightly Agree | Agree | Extremely Agree | **Past behavior**:Please rate to what extent you agree or disagree with below statements (select only one answer) | |
| --- | --- | --- | --- | --- | --- | --- | --- | --- |
|  |  |  |  |  |  |  | In the last year I had experience with HTC services? | Q57 |

| I do not know | No | Yes | Knowledgeabout HIV and HTC services | |
| --- | --- | --- | --- | --- |
| 🞎 | 🞎 | 🞎 | Healthy looking person can be infected with HIV | Q58 |
| 🞎 | 🞎 | 🞎 | HIV infection can be prevented by using a condom | Q59 |
| 🞎 | 🞎 | 🞎 | HTC is important for TB and suspect TB patients | Q60 |
| 🞎 | 🞎 | 🞎 | HTC is provided at TB facilities | Q61 |
| 🞎 | 🞎 | 🞎 | HIV infection transmitted through sexual intercourse | Q62 |

[Thank the participant for his time]
